# Effects of irrigation frequency on root growth, nutrients accumulation, yield, and water use efficiency of *Panax notoginseng* under micro-sprinkler irrigation

**DOI:** 10.3389/fpls.2025.1549506

**Published:** 2025-02-18

**Authors:** Hui Huang, Yuzhe Shi, Anrong Luo, Yanan Xiao, Jiaping Liang, Zijian He

**Affiliations:** ^1^ Xi’an TieYi BinHe High School, Xi’an, China; ^2^ Faculty of Modern Agricultural Engineering, Kunming University of Science and Technology, Kunming, China; ^3^ Yunnan Phosphate Chemical Croup Co., Ltd., National Engineering and Technology Center for the Development & Utilization of Phosphorous Resources, Kunming, China; ^4^ State Key Laboratory of Efficient Utilization of Agricultural Water Resources, Beijing, China; ^5^ Center for Agricultural Water Research in China, China Agricultural University, Beijing, China

**Keywords:** irrigation frequencies, nutrient accumulation, *P. notoginseng*, yield, water use efficiency

## Abstract

Micro-sprinkler irrigation has been a promising irrigation method to promote *Panax notoginseng* (Burk) F. H. Chen production but their scientific irrigation frequency in improving yield and water use efficiency of *P. notoginseng* remains contradictory and inconclusive. The objective of this study was therefore to examine and propose a scientific irrigation frequency in water management of *P. notoginseng* cultivation considering their impact on soil water, soil available nutrients, root growth, yield, and water use efficiency (WUE). The micro-sprinkler irrigation experiment under shading and rain-shelter conditions was carried out in the growing season of *P. notoginseng* from 2017 to 2018.The treatments included four micro-sprinkler irrigation frequencies, such as IF1 (irrigation once every three days), IF2 (irrigation once every five days), IF3 (irrigation once every seven days), and IF4 (irrigation once every nine days) in 2017-2018. The results indicated that the IF3 treatment significantly increased the nitrogen accumulation of *P. notoginseng* (271.98 mg plant-1). In addition, the IF2 treatment enhanced the phosphorus accumulation (27.82 mg plant-1), potassium accumulation (408.38 mg plant-1), total root surface area (67.49 cm2 plant-1), total root volume (3.79 cm3 plant-1) and yield (702 kg ha-1). The IF2 treatment significantly increased WUE by 29.2%, 28.1%, and 37.7% compared with the IF1, IF3, and IF4 treatments, respectively. Our findings suggested that IF2 treatment increased the soil water content, reduced the soil nutrient content, increased the accumulation of phosphorus and potassium in *P. notoginseng*, promoted the root growth of *P. notoginseng*, and improved the quality and yield of *P. notoginseng*, providing a scientific theoretical basis for reasonable water control and green quality production in the cultivation of *P. notoginseng* under shade and rain shelter cultivation.

## 1 Introduction

*P. notoginseng* (Burk. F. H. Chen) was a traditional perennial precious herb with an artificially cultivated history of more than 400 years (Armstrong, 2005; Bengough et al., 2011). It was widely distributed in the world, especially in southwest China (Wang et al., 2006). The roots, stems and leaves of *P. notoginseng* contain saponins, salicylidine, GABA (γ-aminobutyric acid), and other components, which had extremely high pharmacological effects (Li et al., 2022; Zhou et al., 2020). Modern studies confirmed that *P. notoginseng* has multiple pharmacological functions on antihypertensive, antithrombotic, antioxidation and neuroprotective actions, regulating the immune system and promoting metabolism and so on (Yang et al., 2015). *P. notoginseng* was also the main component of Yunnan Baiyao, Compound Danshen Dripping Pills, Pien Tze Huang and many other traditional Chinese medicine preparations, which had important economic value (Ji et al., 2022). Due to its unique medicinal and edible value, the market demand of *P. notoginseng* was growing rapidly (Chen et al., 2023). To improve the yield of *P. notoginseng* and pursue the maximization of economic benefits, growers often use excessive water and fertilizer applications (Xia et al., 2016). However, adequate irrigation was not sustainable for the semiarid areas of Yunnan Province. Excessive irrigation may lead to soil salinization and water resource depletion, which would have a negative impact on the ecological environment and the sustainable development of agricultural management. Therefore, it was urgent to balance the relationship between agricultural production demand and ecological protection in irrigation management, explore the demand of *P. notoginseng* for nutrients and water, and propose appropriate irrigation strategies.

Water and fertilizer regulation was crucial to the physiological growth and nutrient absorption of *P. notoginseng* (Shen, 2024). Irrigation mechanisms could regulate soil water and temperature, and soil water content directly affects plants’ water regimes and is closely related to plants’ healthy growth (Man, 2014). Excessive or low soil water content could significantly affect the growth, water absorption, and yield of *P. notoginseng*, thus reducing water use efficiency (Li et al., 2019). Irrigation requirements vary according to location, soil type and crop growth characteristics, involving the form of irrigation water entering the soil in the root zone of the crops (Lebourgeois et al., 2010; Ravikumar et al., 2011; Rodrigues et al., 2013). Micro-sprinkler irrigation was an effective water-saving irrigation technology, that was widely used in crop precision irrigation and could achieve sustainable economic benefits of crops. Compared with drip irrigation, micro-sprinkler irrigation significantly increased crop yield by 7.3% (Majumder et al., 2008). Appropriate micro-sprinkler irrigation could improve water use efficiency, enhance soil permeability, reduce soil water loss, and effectively increase the yield of *P. notoginseng* (Kumar et al., 2014; Xu et al., 2022; Zheng et al., 2021). Previous studies have found that different irrigation frequencies have significant effects on crop growth and yield (Liu H. et al., 2019; Souza and Vieira, 2020). At the same time, no irrigation frequency will also change the root water absorption capacity of crops (Brighenti, 2024). Adopting scientific and reasonable irrigation methods was of great significance for improving the growth environment of *P. notoginseng* and promoting the sustainable development of *P. notoginseng* cultivation (Yang et al., 2023).

Suitable irrigation enhanced plant immunity and promotes growth by affecting plant nutrient accumulation and soil physical properties, which was beneficial for higher yields (Chai et al., 2016). The water-demand characteristics of many crops have been studied in conventional agricultural cultivation (Kresović et al., 2016; Chai et al., 2016), however, there were few research reports on the effects of irrigation frequencies on *P. notoginseng*. This experiment investigated the effects of micro-sprinkler irrigation combined with organic fertilizer on the growth, root hydraulic conductivity, nutrient accumulation, yield, and water use efficiency of 2-year-old *P. notoginseng*. Therefore, this experiment studied the effect of irrigation frequency of micro-sprinkler irrigation on *P. notoginseng* under the condition of shading and rain avoidance, aiming at (1) exploring the effects of irrigation frequencies on soil water content and soil nutrients in the soil layer of 0~40 cm; (2) analyzing the effects of irrigation frequencies on the morphological characteristics of *P. notoginseng* roots, root hydraulic conductivity, nutrient accumulation, yield and water use efficiency; and (3) determining effective irrigation management strategies for *P. notoginseng* cultivation.

## 2 Materials and methods

### 2.1 Details of experimental site

**Figure 1:**
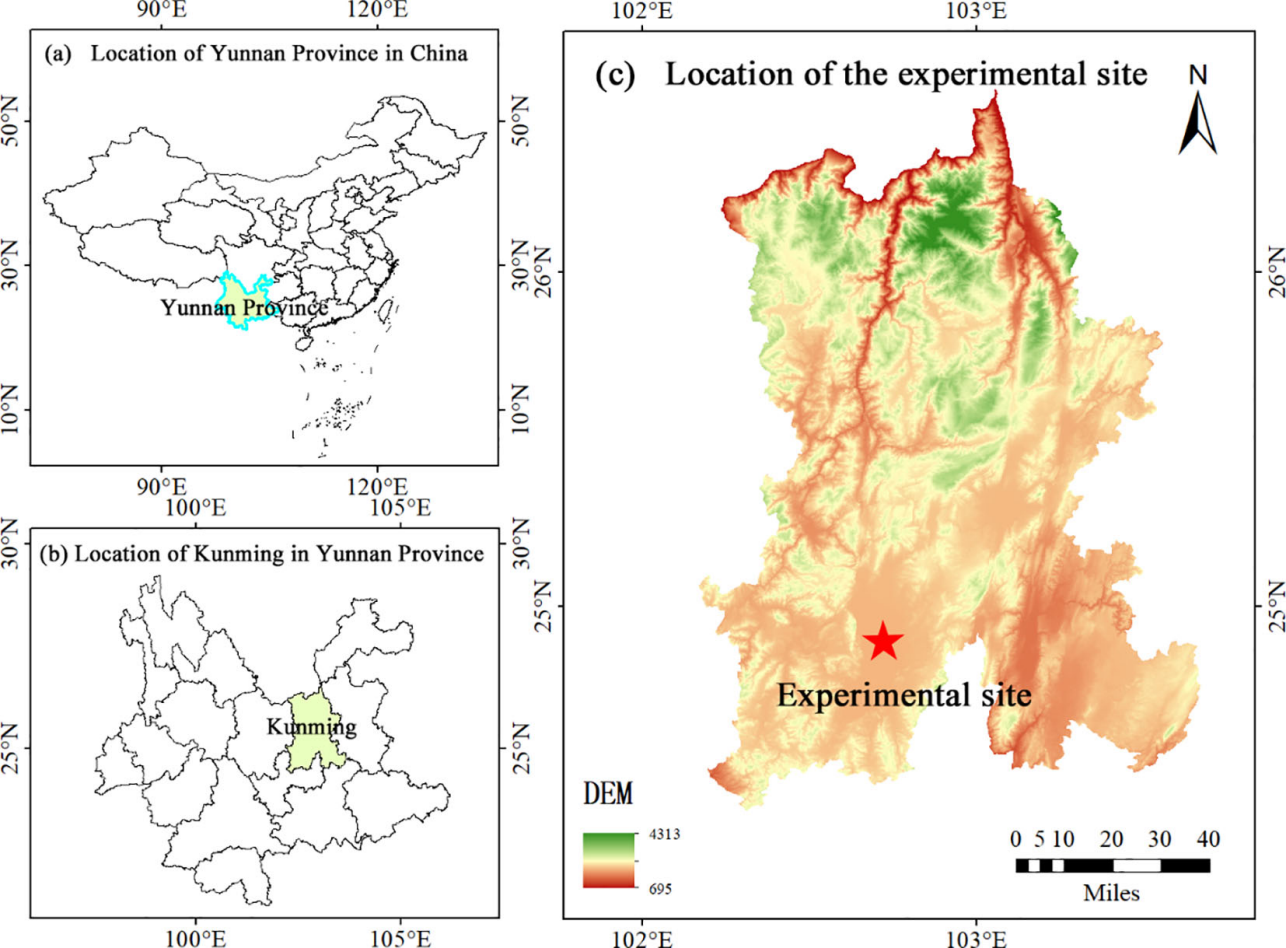
**(A)** The location of Yunnan Province in China. **(B)** the location of Kunming in Yunnan Province. **(C)** The location of the experimental site.

The experiment was conducted in the shade and rain shelter cultivation greenhouse (102°52′13″E, 24°50′57″N) at the experimental base of Kunming University of Science and Technology (KUST) Chenggong Campus from March 2017 to November 2018. The specific geographic location was shown in **Figure 1**. The top of the greenhouse was covered with three layers of shading net, and the light transmittance was 8.3%. The climate was subtropical plateau monsoon. The water used for irrigation was tap water for domestic use, containing trace amounts of sodium chloride and sub-sodium chloride, as well as calcium, magnesium, potassium and sulfate. pH was 6.87, showing weak acidity. Soil properties, both physical and chemical, at the experimental site are shown in **Table 1**.

**Table 1:** The climate and soil profile of the experimental site.

Experimental site	Value
Maximum temperature (°C) Minimum temperature (°C) Mean relative humidity (%) Mean altitude (m) Average annual sunlight (h) Soil pH Soil bulk density (g cm^-3^) Soil field capacity (cm^-3^ cm^-3^) Soil organic matter content (%) Soil total nitrogen content (g kg^-1^) Soil total phosphorus content (g kg^-1^) Soil total potassium content (g kg^-1^) Soil alkali-hydrolyzed nitrogen (mg kg^-1^) Soil ammonium nitrogen (mg kg^-1^) Soil available phosphorus (mg kg^-1^)	28.00 5.00 58.00 1945.00 2200.00 6.00 1.30 0.41 1.32 0.89 0.73 14.08 65.83 21.56 13.80
Soil available potassium (mg kg^-1^)	337.50

### 2.2 Experiment design

The experiment included four irrigation frequency levels (IF_1_:3 d time^-1^, IF_2_:5d time^-1^, IF_3_:7 d time^-1^, IF_4_: 9 d time^-1^). Each treatment was repeated 2 times, a total of 8 experimental cells. In the shade and rain shelter cultivation greenhouse, there were 8 ridges of *P. notoginseng* test field, the ridge length was 4.4 m, the width was 1.2 m, the height was 0.3 m, and the ridge distance was 0.5 m. The experiment used micro-sprinkler fertilization (water and fertilizer integration fertilization technology). The soluble fertilizer (Sichuan Shifang DEMEI company. China) dissolved in water through a proportional fertilization device (Jinhua Yurun Fountain Drip Irrigation Co., Ltd.). China) at a ratio of 1:800. During irrigation, fertilizer is applied simultaneously through a micro-sprinkler irrigation device. Fertilization frequency is 15 days a time. Irrigation and fertilization were conducted from 17:00 to 19:00. During the test, keep the greenhouse ventilated and remove the diseased plants in time.

### 2.3 Determination items and methods

According to previous studies, the root growth of *P. notoginseng* was mainly distributed in the 0-40 cm soil layer (Yue et al., 2023; Zang, 2022), so the soil indexes in this study were sampled and analyzed in 0-40 cm soil layer.

#### 2.3.1 Soil water content

During the experiment, soil samples at depths of 10, 20, 30, and 40 cm were collected by artificial spiral drills at 1 h before irrigation, 1 day after irrigation, and in the middle of irrigation (IF_1_ at 1.5 days after irrigation, IF_2_ at 2.5 days after irrigation, IF_3_ at 3.5 days after irrigation and IF_4_ at 4.5 days after irrigation) at the seedling stage, flowering stage, fruiting stage, and root growth stage, respectively. Determine the soil water content. Each plot was repeated three times to calculate the average soil water content. The soil moisture content of soil samples was determined by the drying method. All soil samples were dried at 105°C for 6-8h to constant weight, and then the soil water content was determined by weighing method. The volumetric soil water content was calculated by multiplying the mass soil water content by the soil bulk density (Liang et al., 2021).

#### 2.3.2 Soil nutrient content

According to the characteristics of the growth stage of *P. notoginseng*, soil samples were collected at seedling, flowering, fruiting, and root growth stages. The sampling depths were 10, 20, 30, and 40 cm, respectively, and each plot was repeated three times. Soil alkali hydrolyzed nitrogen content was determined by the standard method of the Agricultural industry of the People’s Republic of China (LY/T 1229-1999). Soil available phosphorus content was determined by the agricultural industry-standard method of the People’s Republic of China (NY/T 1121.7-2014) and was extracted by ammonium fluorine-hydrochloric acid solution. Soil available potassium content was determined via the flame photometer specified in the agricultural industry-standard method (NY/T 889-2004) (Cheng et al., 2023). The average soil alkali hydrolyzed nitrogen content, available phosphorus, and available potassium were calculated respectively.

#### 2.3.3 *P. notoginseng* nutrients (N, P, K) content

The dried samples of the aboveground and underground parts of *P. notoginseng* were crushed in a grinder and filtered with a 0.5 mm sieve. The total nitrogen content was determined by an automatic Kjeldahl nitrogen analyzer (Haineng Company, China). The content of total phosphorus was determined by the molybdenum antimony anti-colorimetric method. The total potassium content was determined by a flame photometer (Fuentes-Soriano et al., 2019).

#### 2.3.4 Root hydraulic conductivity

Three representative healthy plants were randomly selected from each plot, and the hydraulic conductivity of *P. notoginseng* at seedling, flowering, fruiting, and root growth stages was measured by HPFM-Gen3 high pressure flow meter (Dynamax Company, USA).

#### 2.3.5 Root characteristics, dry matter mass and yield

The aboveground and underground parts of *P. notoginseng* were collected during the root growth stage, each test plot was repeated three times. After the roots were cleaned, the STD4800 root scanner (EPSON, Japan) was used to scan the whole roots. Then the total root length, total root surface area, and total root volume were analyzed and output by the WINRHIZOPRO2007 root analysis system. The aboveground and underground parts of *P. notoginseng* were put into file bags and then dried at 60 °C for 48 hours in the oven (Donnelly et al., 2016). Then the samples were weighed to obtain the dry matter mass of the aboveground and underground parts of *P. notoginseng*.

The yield was calculated by multiplying the average yield per plant by the planting density (number of plants per hectare) and then by the planting area (Xia et al., 2016).

### 2.4 Calculations

#### 2.4.1 Soil water storage and soil water content

Soil water storage (*SWS*) in the root zone (0-40 cm) of *P. notoginseng* was calculated by the following equation (Liang et al., 2019):


(1) $$\begin{array}{c} SWS=10\times ({\bar{\theta }}_{0-10\text{cm}}\times 10+{\bar{\theta }}_{10-20\text{cm}}\times 10+{\bar{\theta }}_{20-30\text{cm}}\times 10\\ +{\bar{\theta }}_{30-40\text{cm}}\times 10) \end{array}$$

Where *SWS* is soil water storage in the root zone (0-40 cm) of *P. notoginseng*, mm; ${\bar{\theta }}_{0-10cm}$ is the average soil water content in 0-10 cm soil layer (cm^3^ cm^-3^); ${\bar{\theta }}_{10-20cm}$ is the average soil water content in 10-20 cm soil layer (cm^3^ cm^-3^); ${\bar{\theta }}_{20-30cm}$ is the average soil water content in 20-30 cm soil layer (cm^3^ cm^-3^); ${\bar{\theta }}_{30-40cm}$ is the average soil water content in 30-40 cm soil layer (cm^3^ cm^-3^).

Soil water content (*SWC*) in the root zone (0-40 cm) of *P. notoginseng* was calculated by the following equation (Liang et al., 2021):


(2) $$\begin{array}{c} SWC=\frac{Fresh\;weigth\;of\;soil-Dried\;weight\;of\;soil}{Dried\;weight\;of\;soil}\\ \times \;100\% \end{array}$$

#### 2.4.2 Evapotranspiration

Evapotranspiration (*ET*) in the root zone (0-40 cm) of *P. notoginseng* was calculated by the following equation (Liang et al., 2019):


(3) ET=P+I+G−D−R−ΔSWS

Where *ET* was soil water consumption in the root zone of *P. notoginseng*, mm; *P* was the precipitation, mm; *I* was water consumption in each plot, mm; *G* is groundwater recharge, mm; *D* was runoff, mm; *R* represented the amount of deep leakage, mm. This experiment was carried out in a rain shelter unaffected by precipitation, groundwater recharge, runoff, and deep leakage. In this experiment, micro-sprinkler irrigation was used for irrigation, timing irrigation. Therefore, the *P*, *G*, *D*, and *R* were neglected. *ΔSWS* is the difference between SWS before and 1 day after irrigation in 0-40 cm soil layer at seedling, flowering, fruiting, and root growth stage.

#### 2.4.3 Water use efficiency

Water use efficiency (WUE) was calculated by the following equation (Liang et al., 2019):


(4) $$WUE=\frac{Y}{ET}$$

Where Y was crop yield, kg ha^−1^; ET was evapotranspiration, mm.

#### 2.4.4 Cost-benefit analysis

Material input mainly includes land leasing costs, labor expenditure, sunshade materials, micro-sprinkler irrigation systems, water and fertilizer integration machinery, *P. notoginseng* seedlings, microbial fertilizers, sunshade nets, plastic tarpaulin renewal and installation, and infrastructure strengthening. The production value was the yield of *P. notoginseng* with a selling price of 96.23 $ kg^-1^ (no pesticides are used in cultivation due to the high saponin content in *P. notoginseng*). Expenditure on land rent, labor, sunshade materials, irrigation, and machinery in 2017 and 2018 were assessed: 1215.25 $ ha^-1^, 50,219.18 $ ha^-1^, 47281.35 $ ha^-1^, 49251.54 $ ha^-1^and 61.52 $,151.21 $, 1126.53 $ and 185.21 $. Inputs of fertilizers were 0.54 $ kg^-1^. Infrastructure development of *P. notoginseng* seedlings and fertilizers, updating and installing shade nets, and installing plastic tarpaulins were 312.42 $ and 1073.42 $. The exchange rate is 1 USD ≈ 6.98 RMB.


(5) $$Net\;profit\left({＄\text{ ha}}^{-1}\right)=\;Total\;revenue-\;Total\;cost$$

### 2.5 Statistical analysis

Microsoft Excel 2018 software was used to sort out the data. SPSS 27.0 was used for the analysis of variance. The analysis of variance was one-way analysis of variance, and the Duncan (*P*< 0.05) method was used to test the difference significance. ArcMap 10.2 was used to map the latitude and longitude profile of the study area, Origin 2022 was used to produce bar charts. Data in the histograms were expressed as mean ± standard deviation (NS not significant, *P< 0.05, **P< 0.01, ***P< 0.001). The Mantel test was run using R software to correlate irrigation frequencies with soil environmental factors, *P. notoginseng* physiological indicators, and yield. Constructing structural equation model (SEM). The Mantel test was used to explore potential associations between irrigation frequencies, soil environmental factors, physiological indicators, and yield of *P. notoginseng*. Pearson’s correlation analysis determined the correlation between soil physicochemical properties and *P. notoginseng* growth parameters. The analysis of these processes was completed through the *ggcor* package in R. Partial Least Squares Path Modelling (*PLS-PM*) was used to investigate the direct or indirect effects of irrigation frequencies, soil water content, and soil nutrients on *P. notoginseng* growth indicators and yield.

## 3 Result

### 3.1 Soil water content and soil water storage

**Figure 2:**
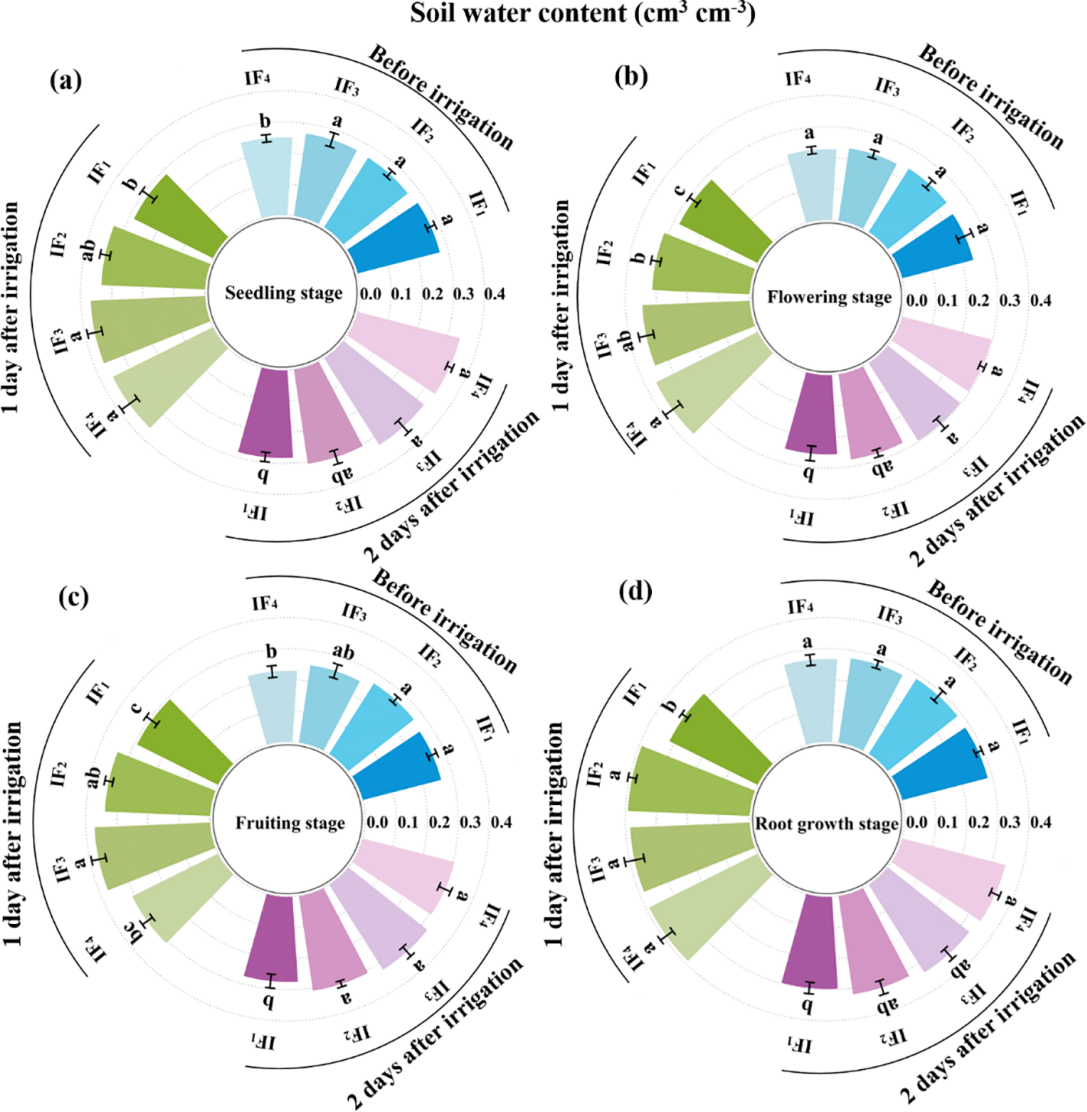
Effects of different irrigation frequencies on soil water content (SWC) in *P. notoginseng* root zone (0-40 cm). **(A)** Soil moisture content at seedling stage. **(B)** Soil water content at flowering stage. **(C)** Soil water content at fruiting stage. **(D)** Soil water content at root growth stage.

The soil water content (SWC) and soil water storage (SWS) in the root zone (0 - 40 cm) of the *P. notoginseng* plant before irrigation and first, second days after irrigation during the seedling stage, flowering stage, and fruiting stage were showed in **Figure 2** and **Table 2**. Soil water content (SWC) decreased with increasing time after irrigation events due to soil evaporation and root water accumulation. According to **Figure 2** and **Table 3**, there were significant (*P<* 0.05) differences in soil water content (SWC) and soil water storage (SWS) at the root zone (0-40 cm soil depth) of *P. notoginseng* among different irrigation frequency treatments. Before irrigation, the order of soil water content (SWC) and soil water storage (SWS) from high to low was IF_1_ > IF_3_ > IF_2_ > IF_4_ at the seeding stage and IF_2_ > IF_1_ > IF_4_ >IF_3_ at other growth stages. Compared with other treatments, the IF_2_ treatment significantly (*P<* 0.05) increased SWC and SWS by 2.4%-6.4% at the flowering stage, 3.0%-17.3% at the fruiting stage, and 0.6%-7.3% at the root growth stage before irrigation. After irrigation, the first and second days SWC and SWS had no significant (*P<* 0.05) regularity in each growth stage. The ΔSWS increased with decreasing irrigation frequencies in the seedling stage, flowering stage, and root growth stage of *P. notoginseng*.

**Table 2:** Effects of different irrigation frequencies on soil water storage (SWS) in the root zone (0-40 cm soil layer) of *P. notoginseng*.

Treatments	Seedling stage			Flowering stage			Fruiting stage			Root growth stage		
Irrigation frequencies	Before irrigation (mm)	After irrigation (mm)	ΔSWS (mm)	Before irrigation (mm)	After irrigation (mm)	ΔSWS (mm)	Before irrigation (mm)	After irrigation (mm)	ΔSWS (mm)	Before irrigation (mm)	After irrigation (mm)	ΔSWS (mm)
IF_1_	108.96 ± 1.60a	121.64 ± 3.01c	12.68 ± 0.31d	94.16 ± 2.35a	112.36 ± 1.97d	18.2 ± 0.32d	104.08 ± 1.56a	116.44 ± 2.49d	12.36 ± 0.26d	113.48 ± 1.35a	126.56 ± 1.57b	13.08 ± 0.18c
IF_2_	105.64 ± 1.37a	133.44 ± 2.20b	27.8 ± 0.4587c	96.44 ± 1.54a	125.32 ± 1.92c	28.88 ± 0.44c	107.24 ± 1.29a	135.28 ± 1.76b	28.04 ± 0.37c	114.21 ± 2.42a	156.62 ± 2.12a	42.41 ± 0.57b
IF_3_	106.76 ± 2.47a	147.2 ± 3.52a	40.44 ± 0.97b	93.96 ± 1.20a	138 ± 3.36b	44.04 ± 1.32b	100.72 ± 2.31b	148.64 ± 3.43a	47.92 ± 1.10a	109.56 ± 1.40b	153.72 ± 4.81a	44.16 ± 1.38b
IF_4_	99.76 ± 1.19b	143.84 ± 4.47a	44.08 ± 1.37a	90.6 ± 1.08b	145.68 ± 4.38a	55.08 ± 1.65a	91.36 ± 1.73c	123.24 ± 2.42c	31.88 ± 0.63b	106.36 ± 1.86b	155.61 ± 3.82a	49.25 ± 1.21a
ANOVE	**	***	***	NS	***	***	***	***	***	**	***	***

The data were the average of three replicates. The mean ± standard deviation within the column, and the different lowercase letters, indicate significant differences between all treatments at the 0.05 level. “NS” means no significance; significant at *P_value_< 0.05; significant at **P_value_< 0.01; significant at ***P_value_< 0.001. IF_1_ represents irrigation frequency once every three days; IF_2_ represents irrigation frequency once every five days; IF_3_ represents irrigation frequency once every seven days. IF_4_ represents irrigation frequency once every nine days.

**Table 3:** The irrigation frequencies (I) and fertilizer rate (F) treatments of *P. notoginseng*.

Treatments		Year	Total irrigation amount (mm)	Total fertilization amount (kg ha^-1^)
Irrigation frequencies	Fertilizer rate			
IF_1_	150 kg ha^-1^	2017	480	150
(3d time^-1^)		2018	510	150
IF_2_	150 kg ha^-1^	2017	480	150
(5d time^-1^)		2018	510	150
IF_3_	150 kg ha^-1^	2017	480	150
(7d time^-1^)		2018	510	150
IF_4_	150 kg ha^-1^	2017	480	150
(9d time^-1^)		2018	510	150

### 3.2 Nutrient

#### 3.2.1 Soil nutrient content

Irrigation frequency significantly affected (*P<* 0.05) the contents of soil alkali-hydrolyzable nitrogen, soil-available phosphorus, and soil-available potassium in the root zone (0-40 cm) of *P. notoginseng* (**Table 4**). The contents of soil alkali-hydrolyzable nitrogen and soil-available potassium in the root zone of *P. notoginseng* decreased first and then increased with the increase of irrigation frequency. The content of soil available phosphorus was IF_4_ > IF_3_ > IF_1_ >IF_2_ at the seedling stage, and IF_3_ > IF_1_ > IF_4_ > IF_2_ at the flowering and fruit stage and root weight gain stage. Under IF_2_ treatment the contents of soil alkali-hydrolyzable nitrogen, available phosphorus, and available potassium were the lowest. Compared with IF_1_, IF_3_, and IF_4_, the IF_2_ treatment significantly (*P<* 0.05) decreased the soil alkali-hydrolyzable nitrogen content by 1.0-9.3%, the soil available phosphorus content by 3.4-11.1%, and the soil available potassium content by 2.1-20.6% at each growth stages. The contents of soil available nitrogen and available potassium in the root zone of *P.notoginseng* under IF_4_ treatment were the highest in each growth stage. Compared with other treatments, the contents of soil available nitrogen and available potassium in IF_4_ treatment were significantly (*P<* 0.05) increased by 0.2-10% and 9.8-23.2%, respectively. Therefore, IF_4_ (9 d time^-1^) treatment was the best irrigation frequency to increase soil available nitrogen content and soil available potassium content.

**Table 4:** Effects of different irrigation frequencies on soil alkali-hydrolyzable nitrogen content, soil available phosphorus content, and soil available potassium content in *P. notoginseng* root zone.

Soil nutrient content	Irrigation	Seedling stage	Flowering stage	Fruiting stage	Root growth stage
Soil alkali hydrolyzed nitrogen content (mg kg^-1^)	IF_1_	72.38 ± 6.48a	64.50 ± 4.39ab	61.80 ± 6.87ab	73.15 ± 3.78a
	IF_2_	71.45 ± 7.19a	60.98 ± 4.63b	57.33 ± 5.65b	67.03 ± 3.85b
	IF_3_	72.15 ± 6.48a	66.95 ± 6.38ab	61.60 ± 7.49ab	73.68 ± 4.28a
	IF_4_	73.83 ± 5.98a	67.10 ± 5.13a	62.10 ± 7.28a	73.85 ± 4.28a
	ANOVE	**	***	***	***
Soil available phosphorus content (mg kg^-1^)	IF_1_	22.51 ± 1.39a	18.21 ± 0.66ab	13.10 ± 0.79ab	20.34 ± 0.92ab
	IF_2_	20.31 ± 0.71b	17.08 ± 0.72c	12.05 ± 0.81b	19.34 ± 0.81b
	IF_3_	21.55 ± 0.65ab	18.70 ± 0.57a	13.56 ± 0.57a	21.15 ± 0.78a
	IF_4_	21.61 ± 0.21ab	17.69 ± 0.56bc	12.63 ± 0.60ab	20.19 ± 0.95ab
	ANOVE	***	***	***	***
Soil available potassium content (mg kg^-1^)	IF_1_	291.75 ± 33.27b	254.02 ± 30.88ab	212.50 ± 26.29ab	187.25 ± 23.90ab
	IF_2_	285.73 ± 29.49c	228.75 ± 33.95b	197.75 ± 20.37b	171.50 ± 22.37b
	IF_3_	294.74 ± 34.83b	253.25 ± 26.53ab	207.25 ± 23.64ab	186.03 ± 23.69ab
	IF_4_	323.75 ± 35.27a	281.25 ± 35.78a	238.75 ± 31.24a	216.01 ± 30.12a
	ANOVE	***	***	***	***

The data were the average of three replicates. The mean ± standard deviation within the column, and the different lowercase letters, indicate significant differences between all treatments at the 0.05 level. “NS” means no significance; significant at *P_value_< 0.05; significant at **P_value_< 0.01; significant at ***P_value_< 0.001. IF_1_ represents irrigation frequency once every three days; IF_2_ represents irrigation frequency once every five days; IF_3_ represents irrigation frequency once every seven days. IF_4_ represents irrigation frequency once every nine days.

#### 3.2.2 Accumulation of nutrient

The effects of different irrigation frequencies on the accumulation of nitrogen accumulation, phosphorus accumulation, and potassium accumulation, in the aboveground part (stem + leaf) and underground part (root) for *P. notoginseng* were significant (*P<* 0.05) (**Table 5**). The accumulation of nitrogen, phosphorus, and potassium in the aboveground part and underground part of *P. notoginseng* increased first and then decreased with the increase of irrigation frequency. The accumulation of nitrogen in the aboveground part and underground part of *P. notoginseng* under the IF_3_ treatment was the largest. The IF_3_ treatment significantly (*P<* 0.05) increased nitrogen accumulation by 3.18% -141.55% compared with IF_1_, IF_2_, and IF_4_. The accumulation of phosphorus and potassium in the aboveground and underground parts of *P.notoginseng* was the largest under IF_2_ treatment. Compared with IF_1_, IF_3,_ and IF_4_, the IF_2_ treatment significant (*P<* 0.05) increased phosphorus accumulation by 17.44% -172.86%, and potassium accumulation by 27.57%-171.68%. The results showed that IF_3_ treatment helped increase the accumulation of nitrogen in *P.notoginseng*, and IF_2_ treatment helped increase the accumulation of phosphorus and potassium in *P.notoginseng*.

**Table 5:** Effects of different irrigation frequencies on the accumulation of nitrogen, phosphorus, and potassium in *P. notoginseng*.

Irrigation Frequencies	Nitrogen accumulation (mg plant^-1^)		Phosphorus accumulation (mg plant^-1^)		Potassium accumulation (mg plant^-1^)	
	Aboveground part	Underground part	Aboveground part	Underground part	Aboveground part	Underground part
IF_1_	61.20 ± 5.33b	132.98 ± 21.64b	4.08 ± 0.09c	12.51 ± 2.51b	64.44 ± 1.69c	209.27 ± 19.59bc
IF_2_	87.44 ± 10.69a	151.43 ± 8.27a	7.34 ± 0.20a	20.48 ± 8.27a	100.55 ± 1.22a	307.82 ± 27.88a
IF_3_	90.22 ± 14.35a	181.76 ± 31.13a	6.25 ± 0.14b	15.66 ± 4.25ab	78.82 ± 3.42b	227.17 ± 18.58b
IF_4_	37.35 ± 6.17c	127.92 ± 20.43b	2.69 ± 0.11d	10.47 ± 1.94b	37.01 ± 2.76d	178.94 ± 19.44c
ANOVE	***	NS	***	NS	***	***

The data were the average of three replicates. The mean ± standard deviation within the column, and the different lowercase letters, indicate significant differences between all treatments at the 0.05 level. “NS” means no significance; significant at *P_value_< 0.05; significant at **P_value_< 0.01; significant at ***P_value_< 0.001. IF_1_ represents irrigation frequency once every three days; IF_2_ represents irrigation frequency once every five days; IF_3_ represents irrigation frequency once every seven days. IF_4_ represents irrigation frequency once every nine days.

### 3.3 Root hydraulic conductivity

The root hydraulic conductivity (*k_r_*) of *P. notoginseng* was significantly (*P<* 0.05) different under different irrigation frequency treatments (**Table 6**). The root hydraulic conductivity (*k_r_*) of *P. notoginseng* increased first and then decreased with the increase of irrigation frequency and the root hydraulic conductivity of *P. notoginseng* treated with IF_2_ was the highest in the seedling stage, fruiting stage, and root growth stage. Affected by different irrigation frequencies, the root hydraulic conductivity (*k_r_*) of *P. notoginseng* under IF_2_ treatment was significantly (*P<* 0.05) increased by 6.69% -19.62% compared with IF_1_, IF_3,_ and IF_4_ treatments. However, the root hydraulic conductivity (*k_r_*) of *P. notoginseng* decreased with the increase in irrigation frequency and the root hydraulic conductivity of *P. notoginseng* treated with IF_4_ was the highest in the flowering stage. The results showed that IF_2_ treatment was the most suitable irrigation frequency to increase the root hydraulic conductivity of *P. notoginseng* during the seedling stage, fruiting stage, and root growth stage of *P. notoginseng*.

**Table 6:** Effects of different irrigation frequency on root hydraulic conductivity of *P. notoginseng*.

Irrigation Frequencies	Root hydraulic conductivity (*k_r_*, 10^-6^ kg m^-1^ Mpa^-1^ s^-1^)			
	Seedling stage	Flowering stage	Fruiting stage	Root growth stage
IF_1_	3.812 ± 0.063c	2.978 ± 0.032c	4.246 ± 0.106b	3.845 ± 0.124c
IF_2_	4.834 ± 0.101a	3.448 ± 0.093b	4.969 ± 0.095b	4.550 ± 0.053a
IF_3_	4.704 ± 0.145a	3.063 ± 0.050c	4.647 ± 0.114a	4.269 ± 0.121b
IF_4_	4.248 ± 0.047b	4.302 ± 0.126a	4.377 ± 0.063b	3.851 ± 0.098c
ANOVE	***	***	***	***

The data were the average of three replicates. The mean ± standard deviation within the column, and the different lowercase letters, indicate significant differences between all treatments at the 0.05 level. “NS” means no significance; significant at *P_value_< 0.05; significant at **P_value_< 0.01; significant at ***P_value_< 0.001. IF_1_ represents irrigation frequency once every three days; IF_2_ represents irrigation frequency once every five days; IF_3_ represents irrigation frequency once every seven days. IF_4_ represents irrigation frequency once every nine days.

### 3.4 Root morphological characteristics

**Figure 3:**
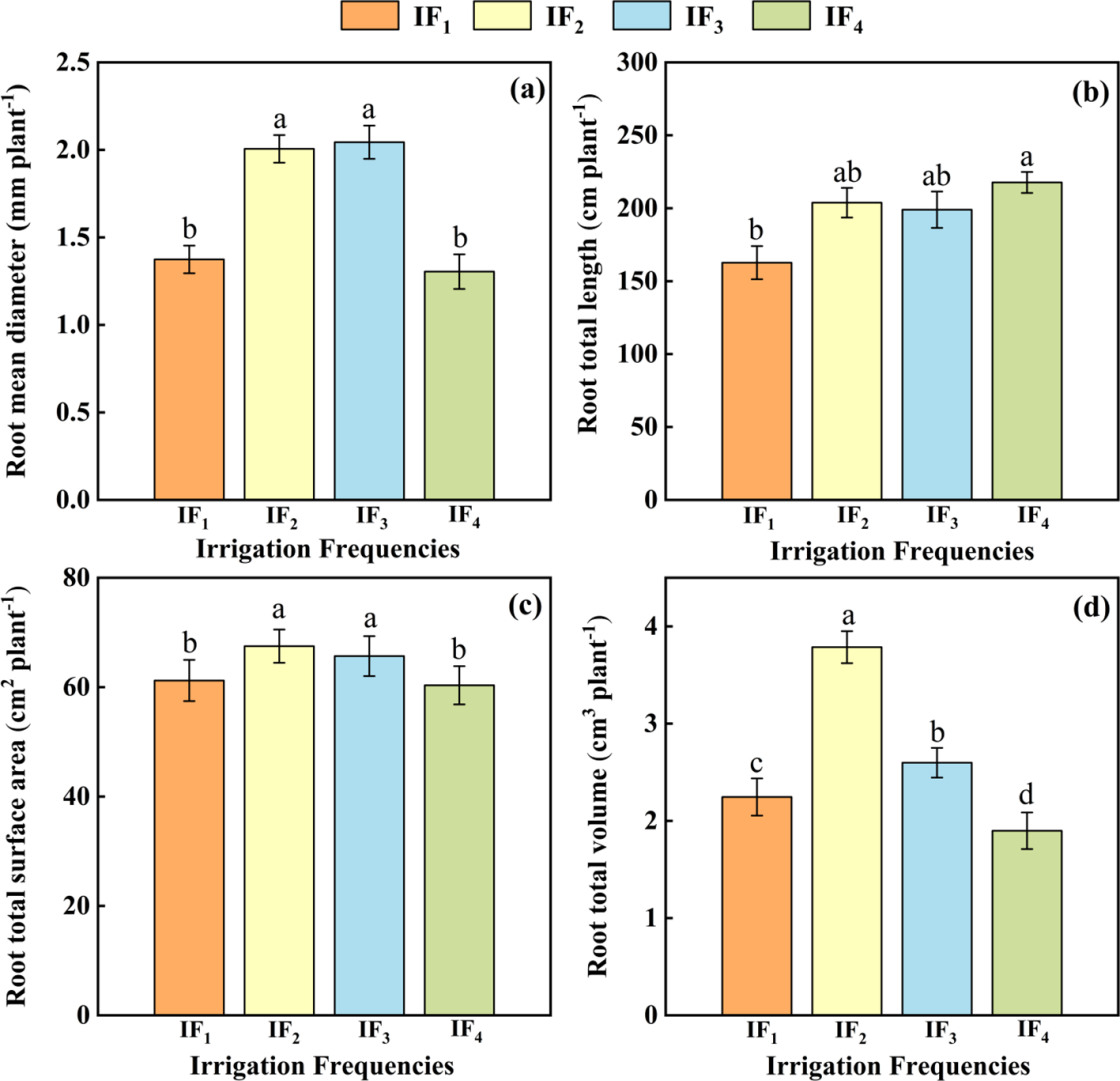
**(A)** Effects of different irrigation frequencies on the root mean diameter of *P. notoginseng*. **(B)** Effects of different irrigation frequencies on the root total length of *P. notoginseng*. **(C)** Effects of different irrigation frequencies on the root total surface area of *P. notoginseng*. **(D)** Effects of different irrigation frequencies on the root total volume of *P. notoginseng*.

The root mean diameter, root total length, root total surface area, and root total volume of *P. notoginseng* were significantly (*P<* 0.05) affected by different irrigation frequencies (**Figure 3**). The average diameter of *P. notoginseng* from high to low was IF_3_ > IF_2_ > IF_1_ > IF_4,_ the root total length of *P. notoginseng* from high to low was IF_4_ > IF_2_ > IF_1_ > IF_3_, the root total surface area, and root total volume of *P. notoginseng* from high to low were IF_2_ > IF_3_ > IF_1_ > IF_4_. The highest average root diameter was 2.04 mm under the IF_3_ treatment. Compared with other treatments, IF_3_ significantly (*P<* 0.05) increased the average root diameter by 1.89%-56.67%(**Figure 3A**). The maximum root total length of *P. notoginseng* was 217.65 cm under the IF_4_ treatment (**Figure 3B**). Compared with other treatments, IF_4_ treatment significantly (*P<* 0.05) increased the root total length by 1.89% -56.67%. Under the IF_2_ treatment, the total root surface area and total root volume of *P. notoginseng* reached the maximum values of 67.49 cm^2^ and 3.79 cm^3^ (**Figures 3C, D**) and the IF_2_ significantly (*P<* 0.05) increased root surface area by 2.76%-11.86% and root volume by 45.77%-99.47% compared with other treatments.

### 3.5 Yield and economic benefits of *P. notoginseng*

**Figure 4:**
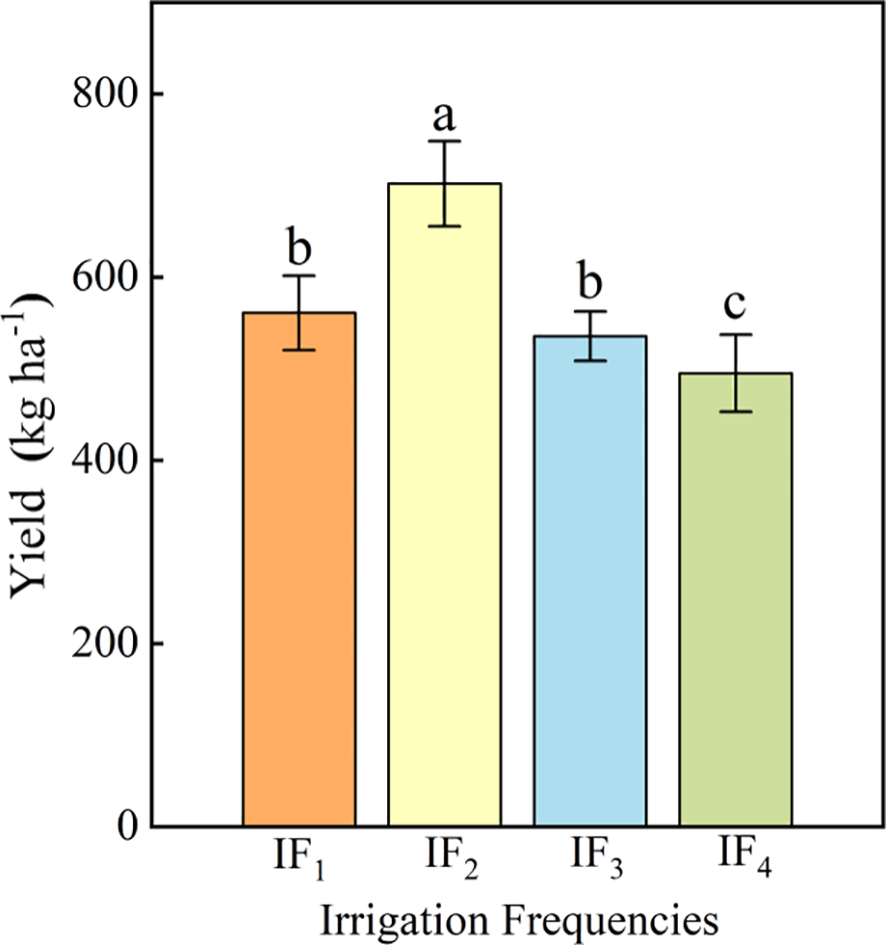
Effects of different irrigation frequencies on the yield of *P. notoginseng.* The data were the average of three replicates. The mean ± standard deviation within the column, and the different lowercase letters, indicate significant differences between all treatments at the 0.05 level. IF_1_ represents irrigation frequency once every three days; IF_2_ represents irrigation frequency once every five days; IF_3_ represents irrigation frequency once every seven days. IF_4_ represents irrigation frequency once every nine days.

The effect of different irrigation frequencies on the yield of *P. notoginseng* was significant (*P<* 0.05) (**Figure 4**). With the increase of irrigation frequency, the yield increased first and then decreased, and reached the highest point under IF_2_ treatment. The yield of *P. notoginseng* was 702 kg ha^-1^ under IF_2_ treatment. Compared with IF_1_, IF_3,_ and IF_4_ treatments, IF_2_ treatment significantly (*P<* 0.05) increased by 25.13%-41.82%. The above results showed that the IF_2_ treatment (5 d time^-1^) had the most significant (*P<* 0.05) effect on increasing the yield of *P. notoginseng*. In order to determine the most suitable irrigation frequency for *P. notoginseng* production, the economic benefits of each treatment were evaluated. Although there was no significant (*P<* 0.05) difference in cost under different irrigation frequency treatments, there were significant (*P<* 0.05) differences in income and economic benefits among treatments. Under the IF_2_ treatment, the income and economic benefits of Panax notoginseng were the highest. Compared with other treatments, the economic benefits of the IF_2_ treatment increased by 41.66-75.96% in 2018. This showed that the IF2 treatment effectively improves the economic benefits. Therefore, it was recommended that local farmers adopt IF2 treatment to improve the yield and economic benefits of *P. notoginseng*.

### 3.6 WUE

Different irrigation frequency treatments significantly affected the water use efficiency (WUE) of *P. notoginseng* (**Table 7**). The water use efficiency of the four treatments from high to low was IF_2_>IF_3_>IF_1_>IF_4_. Among them, the WUE under IF_2_ treatment was significantly increased by 29.2%, 28.1%, and 37.7%, respectively, compared with IF_1_, IF_3,_ and IF_4_ treatments. These results indicated that IF2 treatment (5 d time^-1^) could improve the water use efficiency of *P. notoginseng*.

**Table 7:** Effects of different irrigation frequency treatments on WUE of *P. notoginseng*.

Irrigation Frequencies	Root dry matter mass (g plant^-1^)	Yield (kg ha^-1^)	Evapotranspiration (mm)	Water use efficiency (kg ha^-1^ mm^-1^)
IF_1_	7.48 ± 0.54b	561 ± 40.5b	495.92 ± 0.27a	1.13 ± 0.01b
IF_2_	9.36 ± 0.62a	702 ± 46.5a	478.22 ± 0.46b	1.46 ± 0.17a
IF_3_	7.14 ± 0.36b	535.5 ± 27.1b	465.86 ± 1.19c	1.14 ± 0.01b
IF_4_	6.6 ± 0.56b	495 ± 42.2b	464.93 ± 1.165c	1.06 ± 0.01b
ANOVE	***	***	***	**

The data were the average of three replicates. The mean ± standard deviation within the column, and the different lowercase letters, indicate significant differences between all treatments at the 0.05 level. “NS” means no significance; significant at *P_value_< 0.05; significant at **P_value_< 0.01; significant at ***P_value_< 0.001. IF_1_ represents irrigation frequency once every three days; IF_2_ represents irrigation frequency once every five days; IF_3_ represents irrigation frequency once every seven days. IF_4_ represents irrigation frequency once every nine days.

### 3.7 Analysis of relationship

**Figure 5:**
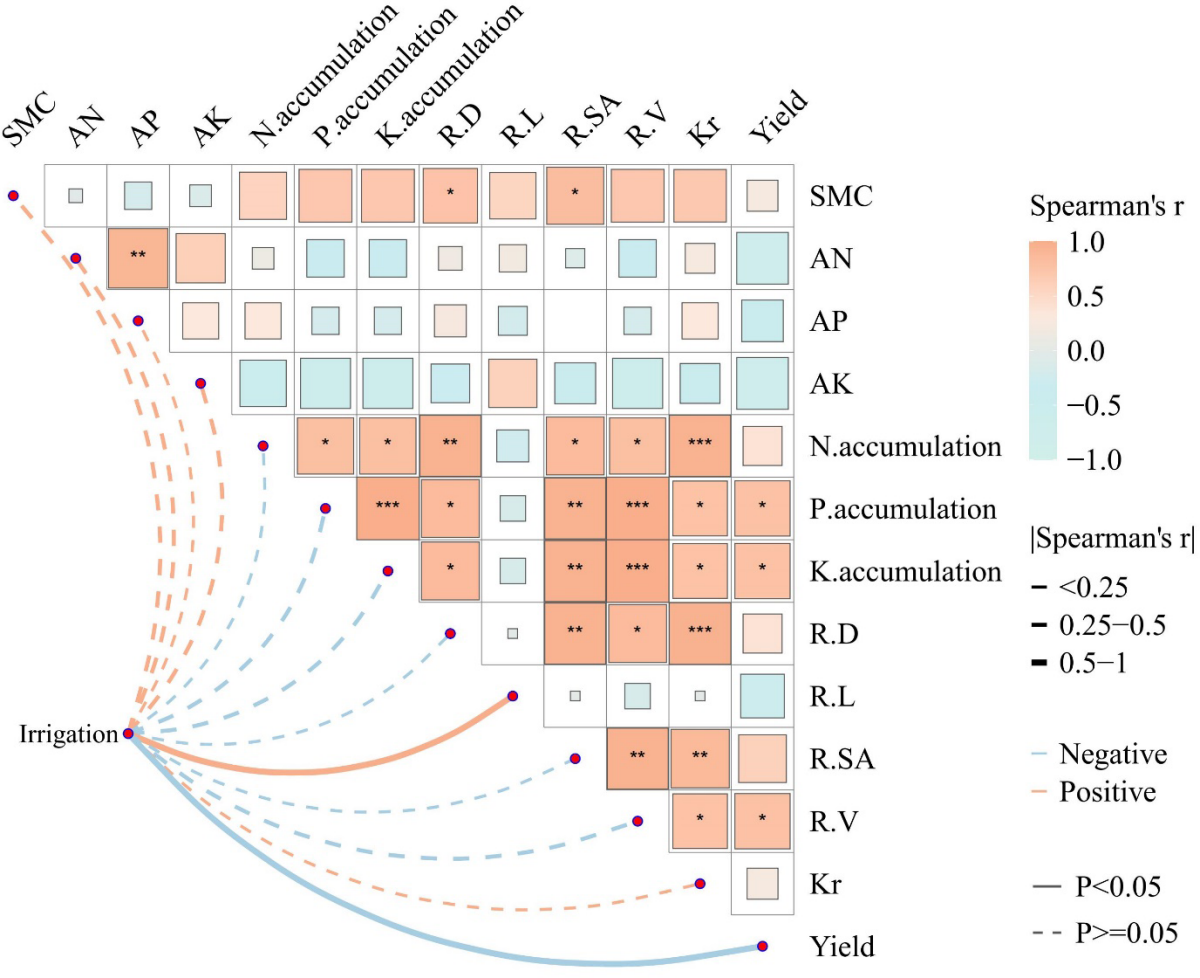
Analysis of the correlation analysis between soil physicochemical properties and the *P. notoginseng* growth indexes under different irrigation frequencies. Pairwise comparisons of the analyzed factors are displayed with a color gradient denoting Spearman’s correlation coefficients. Irrigation frequencies was related to most variables by Mantel test. SMC, soil moisture content; AN, soil alkali hydrolyzale nitrogen content; AP, soil available phosphorus content; AK, soil available potassium content; N-accumulation, *P. notoginseng* nitrogen accumulation; P-accumulation, *P. notoginseng* phosphorus accumulation; K-accumulation, *P. notoginseng* potassium accumulation; R-D, root mean diameter of *P. notoginseng*; R-L, root total length of *P. notoginseng*; R-SA, root total surface area of *P. notoginseng*; R-V, root total volume of *P. notoginseng*; *k_r_*, root hydraulic conductivity of *P. notoginseng*; Yield, yield of *P. notoginseng*. The mean ± standard deviation within the column, and the different lowercase letters, indicate significant differences between all treatments at the 0.05 level. “NS” means no significance; significant at *P_value_< 0.05; significant at **P_value_< 0.01; significant at ***P_value_< 0.001.

The relationship between irrigation frequency and soil environmental factors and physiological indexes and yield of *P. notoginseng* was investigated by Mantel test and Spearman correlation analysis (**Figure 5**). Irrigation frequency showed significant positive correlations with soil moisture content SMC, alkaline dissolved nitrogen content AN, effective phosphorus AP, effective potassium AK, total surface area of *P. notoginseng* root system R-SA, root hydraulic conductivity *k_r_*, and dry matter mass, and irrigation frequency showed significant positive correlations with soil environmental factors and physiological indicators of *P. notoginseng* yield (**Figure 5**). N-accumulation, P-accumulation, K-accumulation, R-D, R-L, and R-V. The frequency of irrigation showed a significant negative correlation with N-accumulation, P-accumulation, K-accumulation, R-D, R-L, and R-V of *P. notoginseng*.

### 3.8 Structural equation model

**Figure 6:**
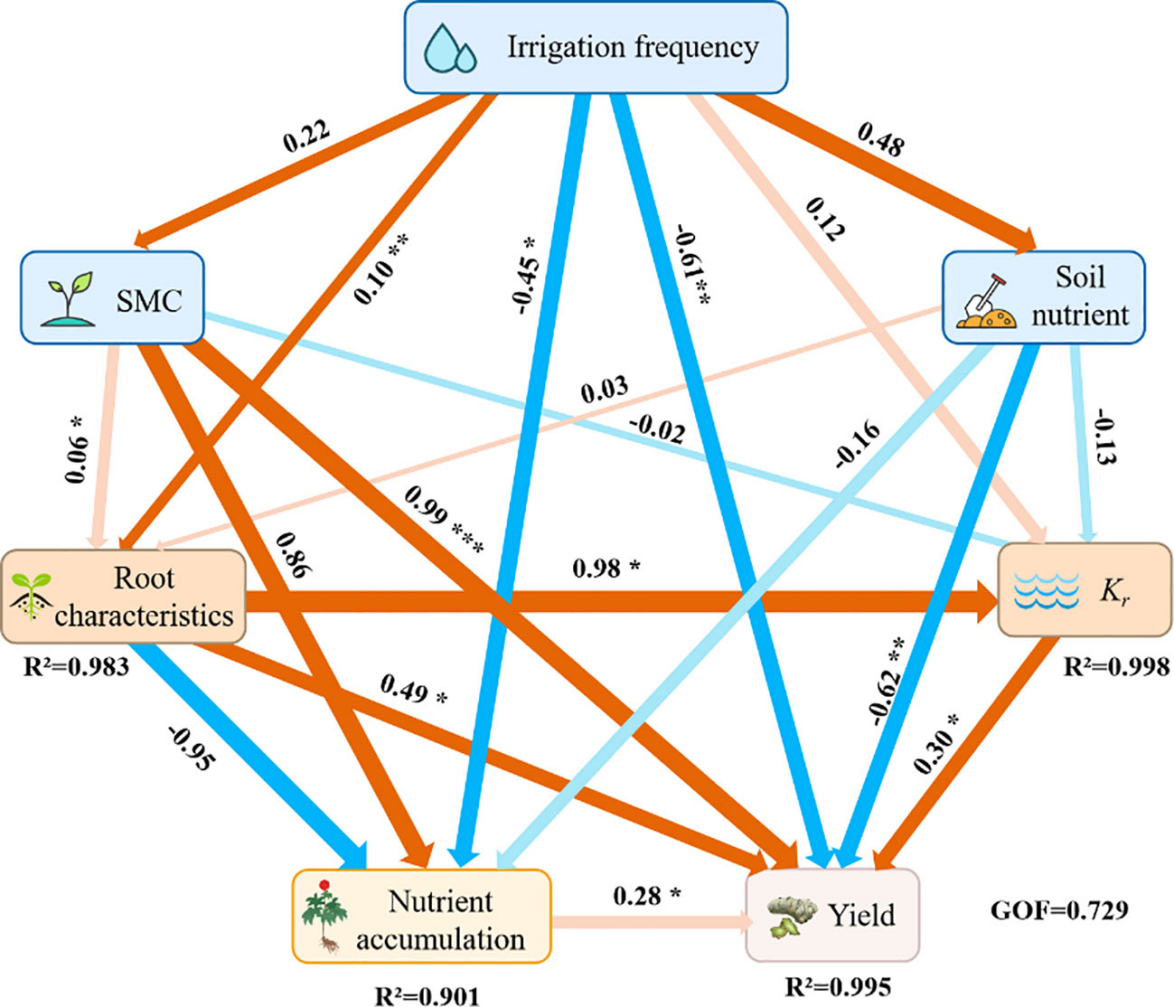
Partial least squares path modeling (*PLS-PM*) shows the direct and indirect effects of irrigation frequency, SMC, and soil nutrients on physiological indicators and yield of *P. notoginseng*. Each box represents a set of variables. Orange and blue arrows indicate positive and negative flows of causality, respectively. The higher path coefficients are shown as bold arrows. ‘*’, ‘**’ and ‘***’ denote significant differences at *P*< 0.05, *P*<0.01, *P*<0.001, respectively.

In this study, irrigation frequency, soil moisture content, and soil nutrients were taken as influencing factors, and a structural equation model was established for their effects on root characteristics, root water conductivity, nutrient accumulation, and yield of *P. notoginseng* (**Figure 6**). Irrigation frequency, soil moisture content, soil nutrients, root characteristics, root water conductivity and nutrient accumulation of *P. notoginseng*. directly or indirectly affected the yield. Among them, root characteristics (0.49, contribution rate: 24.01%) and nutrient accumulation (0.30, contribution rate: 9.0%) had significant positive effects on the yield of *P. notoginseng* (*P*< 0.05), and soil water content (0.9, contribution rate: 81.0%) had a more significant positive effect on the yield of *P. notoginseng* (*P<* 0.01). Root hydraulic conductivity (*k_r_*) had a significant negative effect on the yield of *P. notoginseng* (*P<* 0.05), irrigation frequency (-0.6, contribution rate: 36%), and soil nutrient (-0.2, contribution rate: 4.0%) had a more significant negative effect on the yield of *P. notoginseng* (*P<* 0.01). Meanwhile, according to SEM, it could be found that irrigation frequency, soil moisture content, and soil nutrients significantly increase the yield of *P. notoginseng* by mediating the improvement of root characteristics.

## 4 Discussion

Under the current situation of uneven temporal and spatial distribution of water resources and low utilization efficiency of water resources in Yunnan Province, China, it was of great economic value to research the influence of irrigation frequency on the cultivation of *P. notoginseng*, which provided a sustainable development way to improve the utilization efficiency of water resources and alleviate the uneven distribution of water resources. This study suggested that the appropriate irrigation frequency has the potential to promote the growth and development of *P. notoginseng*, improve the water use efficiency of *P. notoginseng*, and increase the yield and economic benefits of *P. notoginseng*. The findings presented in **Tables 4**–**8**, and **Figures 2**–**6** corroborated these hypotheses.

**Table 8:** Effects of different irrigation frequency treatments on economic benefits of *P. notoginseng*.

Irrigation Frequencies	Cost (×104 $ ha^−1^)	Income (×104 $ ha^−1^)	Economic benefits (×104 $ ha^−1^)
IF_1_	14.95	37.68b	22.73b
IF_2_	14.95	47.15a	32.20a
IF_3_	14.95	35.97b	21.02b
IF_4_	14.95	33.25c	18.30c
ANOVE	NS	***	***

The data were the average of three replicates. The mean ± standard deviation within the column, and the different lowercase letters, indicate significant differences between all treatments at the 0.05 level. “NS” means no significance; significant at *P_value_< 0.05; significant at **P_value_< 0.01; significant at ***P_value_< 0.001. IF_1_ represents irrigation frequency once every three days; IF_2_ represents irrigation frequency once every five days; IF_3_ represents irrigation frequency once every seven days. IF_4_ represents irrigation frequency once every nine days.

### 4.1 Effects of irrigation frequencies on root characteristics of *P. notoginseng*

The root system was the foundation of plant growth, which was not only involved in regulating water balance, nutrient absorption, and growth of plants but also the key organ for plant growth and survival. It is the most direct and earliest part of the plant to feel stress stimulus signals (Li et al., 2021). Root morphological characteristics directly affect plant growth and development, material metabolism, yield, and quality (Lynch, 1995). This study indicated that the root volume and root surface area of *P. notoginseng* root system under medium-frequency irrigation IF_2_ showed a significant increase, but the average diameter of the root system was the largest in the IF_3_ treatment and the maximum total length of the root system under the IF_4_ treatment was largest (**Figure 4**). With this phenomenon, we speculated that the main reason may be that the shallow *P. notoginseng* root system was in a low-oxygen or anaerobic state under IF_1_(high-frequency irrigation) treatment. The morphological characteristics and physiological functions of its root system were greatly affected, and the root growth of *P. notoginseng* was inhibited, which was consistent with the results of previous studies on rice (Bengough et al., 2011; Armstrong, 2005). Under IF_4_ (low-frequency irrigation) treatment, the water content of the surface soil decreased, which stimulated the root system of *P. notoginseng* to penetrate deeper into the soil, and the root length of *P. notoginseng* increased. However, the root diameter did not increase with the growth of the root system. This may be due to the decrease of the turgor pressure of the root cells of *P. notoginseng* caused by the low soil moisture content, and the root growth was blocked (Ahluwalia et al., 2021). At the same time, the cell wall at the distal end of the root was extended, which was a growth strategy adopted by plants to adapt to insufficient water. This helped the roots to continue to grow deeper under drought conditions to obtain more water and nutrients, thereby maximum guaranteeing the water and nutrients required for the healthy growth of *P. notoginseng*. At the same time, this correlation has also been verified in the correlation analysis of each index (**Figure 5**) and the structural equation model (**Figure 6**). Therefore, compared with other treatments, the most suitable treatment for root growth of *P. notoginseng* was IF_2_ treatment.

### 4.2 Effects of irrigation frequencies on nutrient absorption of *P. notoginseng*

Soil nutrient transport and utilization of soil nutrients by plants were affected by various factors (Wang et al., 2023), including water, root absorption capacity, soil physicochemical properties, and soil microorganisms (Briat et al., 2020). Among them, water was the most active and significant factor in soil, and it is an important factor affecting the effective absorption and utilization of nutrients (Wang et al., 2023; Fischer et al., 2019). Inappropriate soil water content could destroy plants’ absorption of water and nutrients, which was not conducive to the normal growth and metabolism of plants (Lyzenga et al., 2023; Xu et al., 2022; Liu L. et al., 2019). In this study, irrigation frequencies significantly affected the distribution of soil water as well as the accumulation and utilization of soil alkali hydrolyzed nitrogen, available phosphorus, and available potassium by *P. notoginseng* roots. The soil water content in the root zone of *P. notoginseng* was the highest under IF_2_ treatment during the fruiting stage and root growth stage. When the irrigation frequency was IF_1_ (3 d time^-1^), soil water was mainly concentrated in the 10 cm-20 cm soil layer due to the low volume but high frequency of irrigation. At the moment, the soil surface water content was too high, which led to poor soil permeability. Soil CO_2_ concentration in the root zone increased and O_2_ content decreased, which was unfavorable for nutrient absorption and utilization by the root system. The low number of irrigations IF_4_ (9 d time^-1^) resulted in mild drought stress, which affected the growth of plant stems, leaves, and roots (Sun et al., 2017; Liu M. et al., 2016). It was detrimental to the accumulation and utilization of soil nutrients by the *P. notoginseng* root system. These results were consistent with Miao and Li’s study (Li et al., 2021; Miao et al., 2015), which found that too high or too low irrigation amount was not conducive to the accumulation of nutrients by *P. notoginseng*.

Soil moisture, soil alkali hydrolyzed nitrogen, available phosphorus, and available potassium content in *P. notoginseng* root zone showed seasonal changes. The trends of soil alkali hydrolyzed nitrogen and available phosphorus were both ‘S’ shaped, with an increasing trend from March 2017 to the seedling stage, a decreasing trend from the seedling stage to the fruiting stage, and an increasing trend from the fruiting stage to the period of the root growth stage. The difference was that the soil alkali hydrolyzed nitrogen decreased the most at the flowering stage, and the soil effective phosphorus content decreased the most at the fruiting stage. It indicated that the peak nitrogen requirement of 2-year-old *P. notoginseng* occurred at the flowering stage and the peak phosphorus requirement occurred at the fruiting stage. These results were similar to those reported by Cui Xiuming (Cui et al., 1994). *P. notoginseng* was a plant with a high demand for potassium, and the soil available potassium content showed a decreasing trend during the whole growth stage. The absorption of K played a key role in the growth of *P. notoginseng* taproots (Wei et al., 2020). The results indicated that the critical nitrogen requirement period of 2-year-old *P. notoginseng* was the flowering stage, the critical phosphorus requirement stage was the fruiting stage, and the critical potassium requirement was the root growth stage. The N, P, and K accumulations under IF_2_ (5 d time^-1^) and IF_3_ (7 d time^-1^) treatments were significantly higher than those under IF_1_ and IF_4_ treatments. Previous studies have shown that appropriate soil water content is more conducive to the propagation of soil microorganisms, enhances the activity of beneficial microorganisms in the soil, accelerates the decomposition of organic matter and nutrient cycling, and promotes the respiration of crop roots, thus facilitating the accumulation of nutrients by the roots of *P. notoginseng* (Zhu et al., 2019). In this study, the IF_2_ treatment had a better effect on the nutrient absorption of *P. notoginseng*.

### 4.3 Effects of irrigation frequencies on root hydraulic conductivity, yield, and WUE of *P. notoginseng*

Plant hydraulic conductivity (*k_r_*), also known as plant water conduction, indicates the water flux of plants under a unit pressure gradient and reflects the ability of roots to absorb and conduct water. It was one of the important water physiological indexes to evaluate the suitability of plant growth (Martre et al., 2001). Plant water conduction was related to soil water content, nutrients, and plant growth stage (Cai et al., 2022). The results of this experiment showed that compared with other treatments, IF2 treatment was the most suitable irrigation frequency to improve the root hydraulic conductivity of *P. notoginseng* at the seedling stage, fruiting stage, and root growth stage (**Figure 4**). This result may be due to the following aspects: (1) The roots of *P. notoginseng* are mainly distributed in the range of 0-20 cm soil layer. When the irrigation frequency was too high (the IF_1_ treatment), the soil surface water content was too high, and then the soil permeability became worse, the root respiration was weakened, the soil CO_2_ concentration in the root zone increased, and the O_2_ content decreased, which led to the change of root morphology and structure, accelerates the senescence and death of root cells, and reduces the water absorption and water conductivity of *P. notoginseng* roots (Vandeleur et al., 2005). (2) When under low-frequency irrigation (IF_4_ treatment), due to the long irrigation cycle, the temperature in the greenhouse is higher, the humidity is lower, and the root system is subjected to water stress for a long time, which leads to the lower root hydraulic conductivity of *P. notoginseng* (Comas et al., 2010). Irrigation was an important factor affecting crop WUE and yield. A meta-analysis found that too high or too low irrigation frequency would lead to a decrease in crop WUE and yield (Cavero et al., 2018). In this study, the WUE (**Table 7**) and yield (**Figure 4**) of *P. notoginseng* under the IF_2_ treatment were the highest, but too-low irrigation frequency (IF_1_) and too-high irrigation frequency (IF_4_) would lead to a decrease in WUE and yield, confirming the above view. This was because the appropriate irrigation frequency could provide a suitable soil environment for crop roots, improve the root hydraulic conductivity of crops, increase WUE, and thus increase crop yield (Zhang et al., 2019). However, too high and too low irrigation frequency would put the soil moisture content in an extreme state, resulting in water stress on crops, thereby affecting the WUE of crops, and resulting in a decrease in crop dry matter accumulation and yield (Liu et al., 2016). At the same time, some researchers found that there was a positive correlation between crop yield and *k_r_* (Du et al., 2024), which was consistent with the fact that the *k_r_* (**Table 6**) and yield (**Figure 4**) of the IF_2_ treatment were higher than the other treatments in this study. From the perspective of economic benefits, the cost of water resources consumed by IF_2_ treatment was 66.6% lower than that of IF_1_ treatment, but the WUE of *P. notoginseng* increased by 29.2% and the yield increased by 42.21%. Therefore, the IF_2_ treatment was more suitable as the best irrigation system for water saving and yield increase of *P. notoginseng*. Crop development and production are restricted by water and fertilizer. In this study, only the single variable of irrigation frequency is considered. In the future research, multivariate experiments should be carried out in combination with different fertilizers to achieve a more perfect water and fertilizer management system.

## 5 Conclusion

It was of great significance to research the effects of irrigation frequency on *P. notoginseng* root growth, nutrient absorption, and yield. According to the current study, compared with other irrigation strategies, the IF_2_ treatment significantly increased the hydraulic conductivity of the *P. notoginseng* root system, promoted the growth of the *P. notoginseng* root system, nutrient absorption as well as yield, and significantly improved WUE and yield. Therefore, the IF_2_ (irrigation once every five days) treatment was recommended as a suitable management strategy to improve the physiological growth of *P. notoginseng*, enhance yield and quality, promote efficient and refined cultivation, and improve farmers’ economic benefits.

## Data Availability

The raw data supporting the conclusions of this article will be made available by the authors, without undue reservation.
